# Associations Between Rumination and the Incidence of First‐Episode Major Depressive Disorder in a Sample of Chinese University Students: From a 1‐Year Longitudinal Study

**DOI:** 10.1155/da/5593375

**Published:** 2026-03-31

**Authors:** Ningning Guo, Yi Zheng, Ruixue Xu, Xingmeng Niu, Yan Qin, Hanyun Li, Jianli Wang, Yan Liu

**Affiliations:** ^1^ School of Mental Health, Jining Medical University, Jining, Shandong, China, jnmc.edu.cn; ^2^ Hongqiao Community Health Service Center of Minhang District, Shanghai Medical College, Fudan University, Shanghai, China, fudan.edu.cn; ^3^ School of Public Health, Jining Medical University, Jining, Shandong, China, jnmc.edu.cn; ^4^ Xijing Hospital, Air Force Medical University, Xi’an, Shaanxi, China, fmmu.edu.cn; ^5^ Department of Community Health and Epidemiology, Faculty of Medicine, Dalhousie University, Halifax, Nova Scotia, Canada, dal.ca

**Keywords:** freshmen, longitudinal cohort study, major depressive disorder, rumination, stressful life events

## Abstract

**Background:**

As a core risk factor for major depressive disorder (MDD), rumination has been confirmed to predict the onset of MDD in adolescents significantly. However, its pathogenic effect on first‐episode MDD in university freshmen has not been systematically evaluated. This study aims to investigate the impact of rumination on the incidence of first‐episode MDD among university freshmen.

**Methods:**

In this longitudinal cohort study, 6985 participants without MDD at baseline completed a follow‐up survey 1 year later. The Chinese version of the Composite International Diagnostic Interview 3.0 (CIDI‐3.0) was used to assess MDD. Rumination was measured by the Ruminative Responses Scale.

**Results:**

The incidence of first‐episode MDD among Chinese freshmen was 2.26%. Symptomatic rumination was significantly associated with a 1‐year incidence of MDD (OR = 1.72, 95% CI: 1.33–2.23), controlling for the effects of baseline depressive symptoms and stressful life events.

**Limitations:**

All the information collected in this survey was from the participants’ recall and selection, making recall and reporting biases possible.

**Conclusions:**

Rumination increases the likelihood of first‐episode MDD among Chinese university freshmen. Therefore, mitigating or even avoiding rumination could be beneficial in reducing the risk of MDD among university freshmen.

## 1. Introduction

Major depressive disorder (MDD) is an increasingly serious human mental health challenge faced by health systems around the world and is also one of the leading causes of disability globally. According to statistics, at least 300 million people worldwide suffer from depression [1], affecting individuals of all ages. University students are in a period of accelerated brain development, which may increase their sensitivity to external stressors. This is especially true for freshmen, who not only need to readjust to a new living environment and build new relationships, but also face higher academic demands and other challenges, all of which increase their risk of developing mental disorders. Statistics show that 29% of university students exhibit varying degrees of depressive symptoms [2], with a growing trend. Moreover, the prevalence of MDD among university students is higher than in the general population [3–5]. Therefore, preventing the occurrence of MDD in college students is a preferred strategy for reducing this significant disease burden. There are many risk factors for MDD, such as rumination. However, research on the impact of rumination on new‐onset MDD among university freshmen is currently limited.

Rumination is a typical repetitive, passive, and relatively uncontrollable negative thinking process [6], closely related to MDD. Patients with MDD exhibit increased levels of rumination [7]. Rumination can prolong the duration and severity of depressive episodes, increase the likelihood of relapse, and intensify negative emotions [8]. In the general population, individuals experiencing a first episode of MDD show higher levels of rumination compared to healthy controls [9]. Adolescents with a history of MDD also report higher levels of self‐reported rumination than healthy controls. A study on adolescents (ages 11–15) found that students with higher levels of rumination are more likely to experience severe depressive episodes in the future, and these episodes may last longer [10]. Evidence suggests that interrupting the rumination process helps minimize the persistence of first‐episode MDD during adolescence [11]. However, these studies mainly focused on cross‐sections or individuals with depressive symptoms and were unable to examine the impact of rumination on new‐onset MDD, especially among college freshmen. Therefore, this study aims to assess the impact of rumination on the risk of first‐episode MDD by analyzing the longitudinal cohort data from Chinese freshmen. To assist in formulating evidence‐based prevention plans and treatment intervention measures for MDD in the university population.

## 2. Methods

### 2.1. Participants

All the participants in this longitudinal cohort study were from the Jining and Rizhao campuses of Jining Medical University and Shandong Second Medical University. The economic development level of the locations where the schools were situated was above average. These students come from different majors and their families live in diverse areas.

### 2.2. Data Collection

A total of 9928 freshmen from the two universities were invited to attend. Among them, 8079 people agreed and completed the baseline survey (T0) from April 2018 to October 2018. After excluding participants with lifetime MDD at the baseline survey, there were still 7476 participants. A total of 6985 cases (93.43%) completed a 1‐year follow‐up investigation (T1) from April 2019 to October 2019. All participants obtained informed consent and signed the informed consent form before participating in the survey. No economic rewards were provided for participation.

Both data collections were carried out using 365 computers in the school library, equipped with an auxiliary investigation system that featured strict logical checks and jump processes. To ensure the orderly progress of the survey, all participants were required to complete it within the prescribed time for their class. Six pretrained investigators were on‐site, ready to answer any questions raised by the participants. To ensure the security and confidentiality of the data, after all participants complete the survey and click the submit button, the survey data were immediately and securely archived on the designated backend server. This study was approved by the Health Research Ethics Committee of Jining Medical University.

## 3. Measures

### 3.1. MDD

MDD is clinically defined as meeting diagnostic criteria for a major depressive episode in the absence of any documented history of manic or hypomanic episodes. In this investigation, MDD was assessed using the Chinese adaptation of the Composite International Diagnostic Interview 3.0 (CIDI‐3.0), which was aligned with DSM‐IV diagnostic frameworks [12]. The CIDI‐3.0 was a validated, fully structured diagnostic interview designed to be administered by trained lay interviewers. Lifetime MDD diagnoses were established at baseline assessment, while 12‐month MDD episodes were identified during the 1‐year follow‐up evaluation. This instrument demonstrated robust psychometric properties for MDD diagnosis, exhibiting 71.1% sensitivity and 89.0% specificity [13]. The CIDI‐3.0 retest reliability was 0.74.

### 3.2. Rumination

The Ruminative Responses Scale (RRS) [14] was used to assess rumination. The RRS consisted of 22 items, which could be divided into three dimensions: symptomatic rumination (1, 2, 3, 4, 6, 8, 9, 14, 17, 18, 19, 22), brooding (5, 10, 13, 15, 16), and reflection (7, 11, 12, 20, 21) [15]. Each item was measured on a scale of 1–4:1 (never) to 4 (always). Finally, all the item scores were added up. The lower the total score, the less rumination. The Cronbach’s *α* of the Chinese RRS, symptomatic rumination, brooding, and reflection were 0.936, 0.902, 0.869, and 0.684 in this study.

### 3.3. Other Measures

Anxiety symptoms were measured at baseline using the Beck Anxiety Inventory (BAI). The scale consists of 21 items, in which answers were provided on a 4‐point scale (such as 1–2–3–4 representing no–mild–moderate–severe). The BAI’s total score is made up of the score for each item. A total BAI score of 45+ was considered to have anxiety [16]. Chinese BAI’s Cronbach’s *α* was 0.95 [17], and it was 0.93 in this survey.

The composition of the Beck Depression Inventory (BDI) [18] was similar to that of BAI. The total score of all item scores when added represented the severity of depression. Clinical severity thresholds were defined as follows: no (0–13), mild (14–19), moderate (20–28), severe (29–63). Chinese BDI’s Cronbach’s *α* was 0.95 [19], and it was 0.91 in this survey.

Adolescent life events in the past year were assessed by the Adolescent Self‐Rating Life Events Checklist (ASLEC). The scale consists of 26 items for which the answers could be “yes” or “no,” and Cronbach’s *α* was 0.84 [20].

Other variables include sex, age, residence (urban or rural), single child (yes or no), school location, student type (undergraduate student or junior university student), major (nonmedicine or medicine), and stressful events in the past year.

### 3.4. Statistical Analysis

In this study, to assess the impact of rumination on the incidence of new MDD within 1 year, participants with lifetime MDD were excluded prior to analysis. The bivariate regression analysis was used to explore the relationship between rumination scores and the risk of MDD. Then, after controlling for some confounding factors, multivariate logistic regression analysis was further used to estimate their correlation. Finally, the receiver operating characteristic (ROC) curve was used to analyze the predictive value of rumination for first‐episode major depression. *p*  < 0.05 represents a significant change.

## 4. Results

### 4.1. Descriptive Analysis

The average age of the participants was 18.36 ± 0.86 years. Table 1 shows the descriptive characteristics of the participants. Among the 6985 participants, 4235 (60.63%) were female, and 2750 (39.37%) were male. There were 6624 (94.80%) undergraduate students and 5133 (73.49%) medicine majors. A total of 4431 (63.44%) students came from rural areas, and 2661 (38.46%) students were only children. The incidence of first‐episode MDD was 2.26% in this freshman cohort. The participants with and without MDD differed significantly in baseline RRS‐symptomatic rumination (*p*  < 0.001), RRS‐brooding (*p*  < 0.001), RRS‐reflection (*p*  < 0.001), and RRS‐total scores (*p*  < 0.001).

**Table 1:** Demographic characteristics of 6985 freshmen of follow‐up.

Variables	New MDD *n*_1_ = 158(%)	No MDD *n*_2_ = 6827(%)	Total	*χ* ^2^/*t*	*p*
Gender					
Male	55 (2.0)	2695 (98.0)	2750	1.41	0.235
Female	103 (2.4)	4132 (97.6)	4235	—	—
Residence					
Urban	59 (2.3)	2495 (97.7)	2554	0.04	0.837
Rural	99 (2.2)	4322 (97.8)	4431	—	—
Student type					
Undergraduate student	156 (2.4)	6468 (97.6)	6624	5.02	0.025
Junior college student	2 (0.6)	359 (99.4)	361	—	—
Major					
Nonmedicine	43 (2.3)	1809 (97.7)	1852	0.04	0.840
Medicine	115 (2.2)	5018 (97.8)	5133	—	—
Single child					
No	97 (2.3)	4161 (97.7)	4258	0.002	0.969
Yes	61 (2.3)	2600 (97.7)	2661	—	—
BDI score^a^					
No (0–13)	127 (1.9)	6440 (98.1)	6567	80.53	<0.001
Mild (14–19)	10 (4.7)	201 (95.3)	211	—	—
Moderate (20–28)	14 (10.6)	118 (89.4)	132	—	—
Severe (29–63)	5 (17.2)	24 (82.8)	29	—	—
BAI score^b^					
<45	149 (2.2)	6648 (97.8)	6783	4.74	0.029
≥45	7 (4.9)	135 (95.1)	156	—	—
Number of stressful events in the past year^c^					
0–3	19 (1.2)	1614 (98.8)	1633	55.21	<0.001
4–6	31 (1.5)	1986 (98.5)	2017	—	—
7–9	33 (1.9)	1664 (98.1)	1697	—	—
≥10	74 (4.6)	1522 (95.4)	1596	—	—
Rumination^d^					
Symptomatic rumination	23.13 ± 6.501	18.86 ± 5.280	6955	−8.175	<0.001
Brooding	11.90 ± 3.313	10.33 ± 2.833	6955	−5.865	<0.001
Reflection	10.83 ± 3.165	9.58 ± 2.709	6955	−4.917	<0.001
Total scores	48.14 ± 12.455	40.75 ± 10.246	6955	−7.379	<0.001

^a^Screened by Beck Depression Inventory (BDI).

^b^Screened by Beck Anxiety Inventory (BAI).

^c^Screened by Adolescent Self‐Rating Life Events Checklist (ASLEC).

^d^Screened by Ruminative Responses Scale (RRS).

The results of the correlation analysis showed a positive correlation between the first‐episode MDD and BDI score, BAI score, number of stressful events in the past year, rumination (total RRS score), symptomatic rumination, brooding, and reflection (Table 2).

**Table 2:** Correlation coefficient matrix between variables.

Variables	Gender	Residence	Student type	Major	Single child	Beck depression score^a^	Beck anxiety score^b^	Number of stressful events in the past year^c^	Rumination^d^	Symptomatic rumination	Brooding	Reflection	New MDD
Gender	1	—	—	—	—	—	—	—	—	—	—	—	—
Residence	0.071^∗^	1	—	—	—	—	—	—	—	—	—	—	—
Student type	0.076^∗^	0.088^∗^	1	—	—	—	—	—	—	—	—	—	—
Major	−0.047^∗^	−0.069^∗^	0.040^∗^	1	—	—	—	—	—	—	—	—	—
Single child	−0.265^∗^	−0.451^∗^	−0.073^∗^	0.051^∗^	1	—	—	—	—	—	—	—	—
Beck depression score^a^	0.013	−0.003	−0.007	−0.045^∗^	0.000	1	—	—	—	—	—	—	—
Beck anxiety score^b^	−0.011	0.005	−0.019	−0.036^∗^	0.003	0.550^∗^	1	—	—	—	—	—	—
Number of stressful events in the past year^c^	0.055^∗^	−0.001	−0.007	−0.083^∗^	−0.008	0.264^∗^	0.270^∗^	1	—	—	—	—	—
Rumination^d^	−0.004	0.014	−0.022	−0.035^∗^	−0.011	0.454^∗^	0.386^∗^	0.331^∗^	1	—	—	—	—
Symptomatic rumination	−0.017	0.013	−0.013	−0.025	−0.006	0.500^∗^	0.427^∗^	0.323^∗^	0.939^∗^	1	—	—	—
Brooding	0.013	0.000	−0.022	−0.048	−0.004	0.371^∗^	0.300^∗^	0.320^∗^	0.898^∗^	0.755^∗^	1	—	—
Reflection	−0.004	0.014	−0.032^∗^	−0.025^∗^	−0.011	0.280^∗^	0.252^∗^	0.227^∗^	0.835^∗^	0.656^∗^	0.720^∗^	1	—
New MDD	0.014	−0.002	−0.027^∗^	−0.002	0.000	0.118^∗^	0.064^∗^	0.081^∗^	0.106^∗^	0.119^∗^	0.081^∗^	0.068^∗^	1

^∗^ *p* < 0.05

^a^Screened by Beck Depression Inventory (BDI).

^b^Screened by Beck Anxiety Inventory (BAI).

^c^Screened by Adolescent Self‐Rating Life Events Checklist (ASLEC).

^d^Screened by Ruminative Responses Scale (RRS).

RRS‐symptomatic rumination (OR = 1.90, 95% CI: 1.67–2.16), RRS‐brooding (OR = 1.65, 95% CI: 1.43–1.91), RRS‐reflection (OR = 1.53, 95% CI: 1.32–1.77), and RRS‐total scores (OR = 1.84, 95% CI: 1.61–2.11) were significantly associated with MDD in bivariate analysis (Table 3). In multivariate analysis, only symptomatic rumination was significantly associated with MDD (OR = 1.72, 95% CI: 1.33–2.23) (Table 4). Furthermore, unlike the effect of symptomatic rumination on the onset of MDD, which did not show significant gender differences, the baseline level of depression has a more significant impact on new‐onset MDD in females (Table 5).

**Table 3:** The univariate logistic regression analyses for first‐episode MDD among 6985 freshmen.

Variables	B	S.E.	Wald *χ* ^2^	OR	95% CI	*p*
Gender (female/male)	−0.20	0.17	1.40	0.82	0.59–1.14	0.236
Residence (rural/urban)	−0.03	0.17	0.04	0.97	0.70–1.34	0.837
Student type (junior college student/undergraduate student)	−0.15	0.71	4.22	0.23	0.06–0.94	0.040
Major (medicine/nonmedicine)	−0.04	0.18	0.04	0.96	0.68–1.37	0.840
Beck depression score^a^	0.44	0.51	76.08	1.56	1.41–1.72	<0.001
Beck anxiety score^b^	0.29	0.06	26.76	1.34	1.20–1.50	<0.001
Number of stressful events in the past year^c^	0.49	0.07	44.26	1.63	1.41–1.88	<0.001
Rumination^d^						
Symptomatic rumination	0.64	0.07	93.53	1.90	1.67–2.16	<0.001
Brooding	0.50	0.07	45.72	1.65	1.43–1.91	<0.001
Reflection	0.42	0.07	32.24	1.53	1.32–1.77	<0.001
Total scores	0.61	0.07	76.93	1.84	1.61–2.11	<0.001

^a^Screened by Beck Depression Inventory (BDI).

^b^Screened by Beck Anxiety Inventory (BAI).

^c^Screened by Adolescent Self‐Rating Life Events Checklist (ASLEC).

^d^Screened by Ruminative Responses Scale (RRS).

**Table 4:** The stepwise logistic regression analyses for first‐episode MDD among 6985 freshmen.

Variables	*β*	S.E.	Wald *χ* ^2^	OR	95% CI	*p*
Rumination^d^						
Symptomatic rumination	0.54	0.13	17.26	1.72	1.33–2.23	<0.001
Brooding	−0.11	0.14	0.62	0.90	0.69–118	0.432
Reflection	−0.03	0.12	0.08	0.97	0.77–1.22	0.776
Sex	0.25	0.17	2.03	1.28	0.91–1.80	0.155
Age	−0.04	0.08	0.23	0.96	0.82–1.13	0.634
Student type	−1.46	0.72	4.15	0.23	0.06–0.95	0.042
Beck depression score^a^	0.27	0.07	13.36	1.31	1.13–1.51	<0.001
Beck anxiety score^b^	−0.11	0.09	1.64	0.90	0.76–1.06	0.895
Number of stressful events in the past year^c^	0.29	0.08	12.77	1.34	1.14–1.58	<0.001

^a^Screened by Beck Depression Inventory (BDI).

^b^Screened by Beck Anxiety Inventory (BAI).

^c^Screened by Adolescent Self‐Rating Life Events Checklist (ASLEC).

^d^Screened by Ruminative Responses Scale (RRS).

**Table 5:** The stepwise logistic regression analyses for new‐onset MDD in male and female students.

Variables	Male			Female			Total		
	*p*	OR	95% CI	*p*	OR	95% CI	*p*	OR	95% CI
Beck depression score^a^	0.310	1.11	0.90–1.37	<0.001	1.36	1.16–1.60	<0.001	1.251	1.100–1.422
Number of stressful events in the past year^b^	0.006	1.37	1.09–1.72	0.05	1.24	1.00–1.53	<0.001	1.306	1.119–1.523
Rumination^c^									
Symptomatic rumination	0.002	1.54	1.18–2.00	<0.001	1.54	1.25–1.90	<0.001	1.535	1.304–1.807

^a^Screened by Beck Depression Inventory (BDI).

^b^Screened by Adolescent Self‐Rating Life Events Checklist (ASLEC).

^c^Screened by Ruminative Responses Scale (RRS).

**Figure 1:**
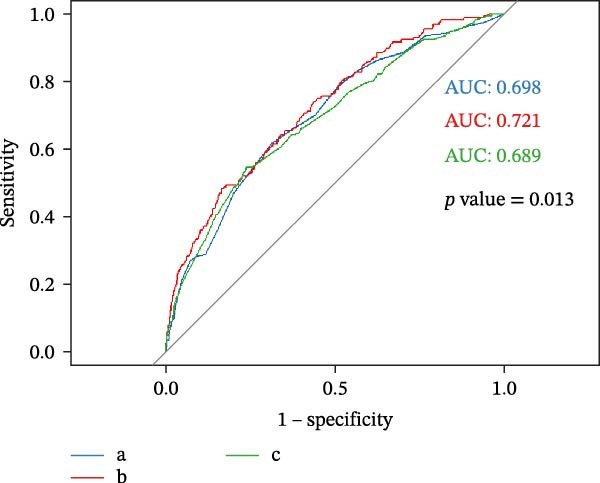
ROC curves of symptomatic rumination in the diagnosis of first‐episode MDD. a: Symptomatic rumination; b: after the introduction of symptomatic rumination; and c: before the introduction of symptomatic rumination. MDD, major depressive disorder; ROC, receiver operating characteristic.

The area under the curve (AUC) for predicting first‐episode MDD using only the symptomatic rumination score was 0.698 (95% CI: 0.656–0.740, *p*  < 0.001), which was significantly higher than the probability. The symptomatic rumination score was introduced into the risk factor model, which included sex, age, student type, Beck depression score, and number of stressful events in the past year. The AUC increased from 0.689 (95% CI: 0.646–0.733, *p*  < 0.001) to 0.721 (95% CI: 0.681–0.761, *p*  < 0.001). The sensitivity and specificity before the introduction of symptomatic rumination were 61.5% and 68.8%, respectively. After the introduction of symptomatic rumination, the sensitivity was 57.1% (fewer real cases were correctly identified), and the specificity was 74.7% (more noncases were correctly identified). A statistically significant difference was observed between the risk model before and after the introduction of symptomatic rumination (*p* = 0.013) (Figure 1).

## 5. Discussion

In this longitudinal cohort study, we found that the incidence of first‐episode MDD was 2.26% among our participants. More importantly, we also found that symptomatic rumination was significantly associated with a 1‐year incidence of MDD (OR = 1.72, 95% CI: 1.33–2.23), controlling for the effects of baseline depressive symptoms and stressful life events.

The lifetime prevalence rate of MDD in the general population is 12% [21]. Among all age groups in the United States, young people aged 18–25 have the highest proportion of suffering from MDD, with a prevalence rate of 18.6% [22]. It has been reported that 5.9% of college students in low‐ and middle‐income countries suffer from MDD each year [23]. In one study, the lifetime prevalence rate of MDD among freshmen was 5.52%, which was higher than 3.90% among the general population in China [24]. This indicates that college students have a higher risk of developing MDD than the general population. A follow‐up survey of freshmen in Western countries such as Belgium (baseline: *n* = 2519, 1‐year follow‐up: *n* = 958) found that the incidence of MDD among freshmen was 6.9% [25], significantly higher than our results. Our results were also slightly lower than those of previous student‐based cross‐sectional surveys on the incidence of MDD among university students (2.4%−4.0%) [5, 26]. There may be several reasons for this difference. First, differences in social economy, geographical location, and cultural background directly affect an individual’s growth environment, resource acquisition, and psychological stress levels. For instance, economic pressure is positively correlated with MDD, which is consistent with previous studies on different demographics and geographical locations [27–29]. Second, Chinese people are reluctant to let others know about their psychological conditions [19]. Third, compared with senior students, freshmen face less pressure in terms of further education, graduation, and employment. Furthermore, differences in investigation methods, the number of interviewees, and other aspects may also be important reasons for the disparity in the incidence of new‐onset MDD among freshmen in China and Western countries.

Rumination plays a central role in the mental health of adolescents and young adults. One study showed that rumination in adolescents could prospectively predict episodes of MDD [30]. Our findings also indicate that rumination is associated with the risk of new‐onset MDD in college freshmen, consistent with previous research. The ROC curve analysis showed that adding symptomatic rumination, the AUC increases, indicating an improvement in the prediction performance of the first onset of MDD during the follow‐up period (Figure 1). Although the sensitivity of the model decreased after adding symptomatic rumination, the specificity significantly increased. The significant increase in specificity (reducing false positives) has unique practical value among college students. It reduces the potential psychological burden on healthy students in the college student population through reducing false positives [31, 32]. For example, labeling a healthy university freshman as “likely to be MDD” (false positive) may bring psychological suggestion and stigma. The inclusion of the symptomatic rumination variable precisely protects the mental health of this group of students by improving specificity, avoiding overmedicalization, and allowing limited campus psychological resources to focus on those high‐risk individuals with both depressive symptoms and symptomatic rumination. Rumination itself is not only a predictor but also an interventional treatment target [33]. Even if the new model misses some people (false negatives), the people it identifies not only know “they are at risk,” but also know that “the risk comes from rumination.” Therefore, conducting “rumination intervention training” (such as cognitive and behavioral therapy [CBT]) for these students makes the intervention pathway clearer and could be more effective. In a randomized controlled trial, CBT focusing on rumination led to a 12% remission rate of depression [34]. Another randomized controlled trial targeting adolescents with MDD found that after cognitive–behavioral intervention, functional MRI scans revealed significant reductions in self‐reported depression and rumination [35]. Increasing research has found that mindfulness‐based cognitive therapy helps patients break this ruminative cycle, transforming it into healthier and more adaptive thinking patterns, thereby alleviating symptoms in MDD patients and preventing relapse [36–38].

Therefore, this study can provide valuable insights into how future research might explore shifting college students’ mental health screening strategies from broad coverage to precision intervention within a hierarchical intervention system.

## 6. Limitations

The current investigation acknowledges several limitations. First, both surveys were conducted in a self‐reported format, so there is recall and selection bias. Second, the sample size of this survey is limited. Consequently, the results may not fully reflect the incidence of MDD among Chinese university students. More accurate data would likely require a nationwide sample survey. Third, these findings may not apply to other models.

## 7. Conclusions

Our findings revealed that symptomatic rumination is a risk factor for new MDD among Chinese university students. Therefore, priority should be given to students’ psychological well‐being, and efforts should be made to reduce or prevent MDD associated with negative rumination.

## Author Contributions

Yan Liu and Jianli Wang were responsible for every process of this research and revised the manuscript. Ningning Guo and Yi Zheng performed data analysis and drafted the manuscript. Ruixue Xu, Xingmeng Niu, Yan Qin, and Hanyun Li conducted the study execution, collected data, and performed quality control checks.

## Funding

This study was supported by the National Natural Science Foundation of China (Grant 81901391), the Taishan Scholars Program of Shandong Province (Grant tsqn20190914), the Research Fund for Helin’s Academician Workstation of New Medicine and Clinical Translation in Jining Medical University (Grant JYHL2021MS25), and the Medical and Health Science and Technology Development Plan of Shandong Province (Grant 202402080726).

## Disclosure

All authors reviewed and approved the final submission.

## Ethics Statement

This study was carried out under the guidance of the Medical Research Ethics Committee of Jining Medical University (2019‐JS‐004) and received the consent of all participants at the same time.

## Conflicts of Interest

The authors declare no conflicts of interest.

## Data Availability

The data that support the findings of this study are available upon request from the corresponding author. The data are not publicly available due to privacy or ethical restrictions.

## References

[1] Guo N. , Wang X. , Xu M. , Bai J. , Yu H. , and Le Zhang , PI3K/AKT Signaling Pathway: Molecular Mechanisms and Therapeutic Potential in Depression, Pharmacological Research. (2024) 206, 10.1016/j.phrs.2024.107300, 107300. 38992850

[2] Gao L. , Xie Y. , Jia C. , and Wang W. , Prevalence of Depression Among Chinese University Students: A Systematic Review and Meta-Analysis, Scientific Reports. (2020) 10, no. 1, 15897–15907, 10.1038/s41598-020-72998-1. 32985593 PMC7522998

[3] Ibrahim A. K. , Kelly S. J. , Adams C. E. , and Glazebrook C. , A Systematic Review of Studies of Depression Prevalence in University Students, Journal of Psychiatric Research. (2013) 47, no. 3, 391–400, 10.1016/j.jpsychires.2012.11.015, 2-s2.0-84873525429. 23260171

[4] Lim G. Y. , Tam W. W. , Lu Y. , Ho C. S. , Zhang M. W. , and Ho R. C. , Prevalence of Depression in the Community From 30 Countries Between 1994 and 2014, Scientific Reports. (2018) 8, no. 1, 2861–2870, 10.1038/s41598-018-21243-x, 2-s2.0-85041945312. 29434331 PMC5809481

[5] Li W. , Meng X. , and Xu Z. , et al.Prevalence, Correlates of Major Depression: A Mental Health Survey Among Undergraduates at a Mainland Chinese University, Asia-Pacific Psychiatry. (2016) 8, no. 3, 206–214, 10.1111/appy.12202, 2-s2.0-84978832743. 26178524

[6] Nolen-Hoeksema S. , Wisco B. E. , and Lyubomirsky S. , Rethinking Rumination, Perspectives on Psychological Science. (2008) 3, no. 5, 400–424, 10.1111/j.1745-6924.2008.00088.x, 2-s2.0-84993784698. 26158958

[7] Zhu X. , Wang X. , and Xiao J. , et al.Evidence of a Dissociation Pattern in Resting-State Default Mode Network Connectivity in First-Episode, Treatment-Naive Major Depression Patients, Biological Psychiatry. (2012) 71, no. 7, 611–617, 10.1016/j.biopsych.2011.10.035, 2-s2.0-84858282936. 22177602

[8] Nolen-Hoeksema S. and Morrow J. , A Prospective Study of Depression and Posttraumatic Stress Symptoms After a Natural Disaster: The 1989 Loma Prieta Earthquake, Journal of Personality and Social Psychology. (1991) 61, no. 1, 115–121, 10.1037/0022-3514.61.1.115, 2-s2.0-0026197625. 1890582

[9] Wang H.-Y. , You H.-L. , and Song C.-L. , et al.Shared and Distinct Prefrontal Cortex Alterations of Implicit Emotion Regulation in Depression and Anxiety: An fNIRS Investigation, Journal of Affective Disorders. (2024) 354, 126–135, 10.1016/j.jad.2024.03.032. 38479517

[10] Abela J. R. Z. and Hankin B. L. , Rumination as a Vulnerability Factor to Depression During the Transition From Early to Middle Adolescence: A Multiwave Longitudinal Study, Journal of Abnormal Psychology. (2011) 120, no. 2, 259–271, 10.1037/a0022796, 2-s2.0-79956026641. 21553940

[11] Park R. J. , Goodyer I. M. , and Teasdale J. D. , Effects of Induced Rumination and Distraction on Mood and Overgeneral Autobiographical Memory in Adolescent Major Depressive Disorder and Controls, Journal of Child Psychology and Psychiatry. (2004) 45, no. 5, 996–1006, 10.1111/j.1469-7610.2004.t01-1-00291.x, 2-s2.0-3242723296. 15225341

[12] Kessler R. C. and Üstün T. B. , The World Mental Health (WMH) Survey Initiative Version of the World Health Organization (WHO) Composite International Diagnostic Interview (CIDI), International Journal of Methods in Psychiatric Research. (2004) 13, no. 2, 93–121, 10.1002/mpr.168, 2-s2.0-4444333309. 15297906 PMC6878592

[13] Huang Y. Q. , Xie S. F. , and Lu J. , et al.Community-Based Evaluation of the Reliability and Validity of Chinese Version of Composite International Diagnostic Interview-3.0, Chinese Mental Health Journal. (2010) 12, no. 1, 21–24.

[14] Nolen-Hoeksema S. , The Role of Rumination in Depressive Disorders and Mixed Anxiety/Depressive Symptoms, Journal of Abnormal Psychology. (2000) 109, no. 3, 504–511, 10.1037/0021-843X.109.3.504, 2-s2.0-0033819572. 11016119

[15] Han X. and Yang H. F. , Chinese Version of Nolen-Hoeksema Ruminative Responses Scale (RRS) Used in 912 College Students: Reliability and Validity, Chinese Journal of Clinical Psychology. (2009) 17, no. 5, 550–551.

[16] Vereecke S. , Sorensen K. , and Zhu J. , et al.The Impact of Physical Conditions on the Incidence of Major Depressive Disorder in Chinese University Students: Results From a Longitudinal Study, Journal of Affective Disorders. (2022) 303, 301–305, 10.1016/j.jad.2022.02.041. 35176340

[17] Cheng S. K. W. , Wong C. S. , and Wong K. C. , et al.A Study of Psychometric Properties, Normative Scores and Factor Structure of Beck Anxiety Inventory Chinese Version, Chinese Journal of Clinical Psychology. (2002) 10, no. 1, 4–6.

[18] Beck A. T. , Ward C. H. , Mendelson M. , Mock J. , and Erbaugh J. , An Inventory for Measuring Depression, Archives of General Psychiatry. (1961) 4, no. 6, 561–571, 10.1001/archpsyc.1961.01710120031004, 2-s2.0-70350654728. 13688369

[19] Cheng J. , Liu D. , and Zheng H. , et al.Perceived Parenting Styles and Incidence of Major Depressive Disorder: Results From a 6985 Freshmen Cohort Study, BMC Psychiatry. (2023) 23, no. 1, 230–239, 10.1186/s12888-023-04712-0. 37020196 PMC10074813

[20] Zheng H. , Liu D. , Cheng J. , Wang D. B. , Liu Y. , and Wu Y. , Negative Life Events Increase the Risk of Suicidal Ideation in 6653 Chinese Freshmen: From a 1-Year Longitudinal Study, Journal of Affective Disorders. (2022) 299, 604–609, 10.1016/j.jad.2021.12.039. 34942231

[21] Pedersen C. B. , Mors O. , and Bertelsen A. , et al.A Comprehensive Nationwide Study of the Incidence Rate and Lifetime Risk for Treated Mental Disorders, JAMA Psychiatry. (2014) 71, no. 5, 573–581, 10.1001/jamapsychiatry.2014.16, 2-s2.0-84900433155. 24806211

[22] Substance Abuse and Mental Health Services Administration , Key Substance Use and Mental Health Indicators in the United States: Results From the 2021 National Survey on Drug Use and Health, 2022, HHS Publication, No. PEP22-07-01-005, NSDUH Series H-57.

[23] Auerbach R. P. , Alonso J. , and Axinn W. G. , et al.Mental Disorders Among College Students in the World Health Organization World Mental Health Surveys, Psychological Medicine. (2016) 46, no. 14, 2955–2970, 10.1017/S0033291716001665, 2-s2.0-84980335897. 27484622 PMC5129654

[24] Huang Y. Q. , Wang Y. , and Wang H. , et al.Prevalence of Mental Disorders in China: A Cross-Sectional Epidemiological Study, The Lancet Psychiatry. (2019) 6, no. 3, 211–224, 10.1016/S2215-0366(18)30511-X, 2-s2.0-85061671000. 30792114

[25] Ebert D. D. , Buntrock C. , and Mortier P. , et al.Prediction of Major Depressive Disorder Onset in College Students, Depression and Anxiety. (2018) 36, no. 4, 294–304, 10.1002/da.22867, 2-s2.0-85058047199. 30521136 PMC6519292

[26] Chen L. , Wang L. , and Qiu X. H. , et al.Depression Among Chinese University Students: Prevalence and Socio-Demographic Correlates, PLoS ONE. (2013) 8, no. 3, 10.1371/journal.pone.0058379, 2-s2.0-84874904429. PMC3596366 23516468

[27] Szkody E. , Hobaica S. , Owens S. , Boland J. , Washburn J. J. , and Bell D. , Financial Stress and Debt in Clinical Psychology Doctoral Students, Journal of Clinical Psychology. (2023) 79, no. 3, 835–853, 10.1002/jclp.23451. 36226891

[28] Zietz S. , Lansford J. E. , and Liu Q. , et al.A Longitudinal Examination of the Family Stress Model of Economic Hardship in Seven Countries, Children and Youth Services Review. (2022) 143, 10.1016/j.childyouth.2022.106661, 106661. 36339096 PMC9631805

[29] Wang F. , Rong L. , and Luo L. , et al.Associations Between Psychological Stress and the Risk of First Onset of Major Depression Disorder: Results From a Longitudinal Study in 6985 Chinese First-Year Students, Psychology Research and Behavior Management. (2024) 17, 3585–3593, 10.2147/PRBM.S482482. 39431158 PMC11491097

[30] Nolen-Hoeksema S. , Stice E. , Wade E. , and Bohon C. , Reciprocal Relations Between Rumination and Bulimic, Substance Abuse, and Depressive Symptoms in Female Adolescents, Journal of Abnormal Psychology. (2007) 116, no. 1, 198–207, 10.1037/0021-843X.116.1.198, 2-s2.0-33847333292. 17324030

[31] Sheehan A. M. and McGee H. , Screening for Depression in Medical Research: Ethical Challenges and Recommendations, BMC Medical Ethics. (2013) 14, no. 1, 4–7, 10.1186/1472-6939-14-4, 2-s2.0-84871951189. 23298315 PMC3556128

[32] Jansen S. N. G. , Mulder B. C. , and Boekhold A. E. , Presymptomatic Screening for Risks to Children’s Mental Health: Ethical Considerations From a European Focus Group Study With Mental Health Professionals, Journal of Bioethical Inquiry. (2025) 10.1007/s11673-025-10473-0. PMC13388338 40952615

[33] Lubbers J. , Geurts D. E. M. , and Lubbers P. S. , et al.Rumination and Self-Compassion Moderate Mindfulness-Based Cognitive Therapy for Patients With Recurrent and Persistent Major Depressive Disorder: A Controlled Trial, Depression and Anxiety. (2024) 2024, no. 1, 10.1155/da/3511703, 3511703. 40226644 PMC11918904

[34] Watkins E. R. , Mullan E. , and Wingrove J. , et al.Rumination-Focused Cognitive-Behavioural Therapy for Residual Depression: Phase II Randomised Controlled Trial, British Journal of Psychiatry. (2011) 199, no. 4, 317–322, 10.1192/bjp.bp.110.090282, 2-s2.0-80053388898. 21778171

[35] Jacobs R. H. , Watkins E. R. , and Peters A. T. , et al.Targeting Ruminative Thinking in Adolescents at Risk for Depressive Relapse: Rumination-Focused Cognitive Behavior Therapy in a Pilot Randomized Controlled Trial With Resting State fMRI, PLoS ONE. (2016) 11, no. 11, 10.1371/journal.pone.0163952, 2-s2.0-84996483810. PMC5120778 27880789

[36] Wang J. , Ren F. , Gao B. , and Yu X. , Mindfulness-Based Cognitive Therapy in Recurrent MDD Patients With Residual Symptoms: Alterations in Resting-State Theta Oscillation Dynamics Associated With Changes in Depression and Rumination, Frontiers in Psychiatry. (2022) 13, 10.3389/fpsyt.2022.818298, 818298. 35321228 PMC8936084

[37] Izabela S. , Mateusz G. , and Marzwna R. , et al.Mindfulness-Based Cognitive Therapy Reduces Clinical Symptoms, but Do Not Change Frontal Alpha Asymmetry in People With Major Depression Disorder, International Journal of Neuroscience. (2021) 131, no. 5, 453–461, 10.1080/00207454.2020.1748621. 32223344

[38] Farb N. , Adam A. , and Arun R. , et al.Prevention of Relapse/Recurrence in Major Depressive Disorder With Either Mindfulness-Based Cognitive Therapy or Cognitive Therapy, Journal of Consulting and Clinical Psychology. (2018) 86, no. 2, 200–204, 10.1037/ccp0000266, 2-s2.0-85038617003. 29265831

